# The Effect of a Synthetic Heparan Sulfate on the Healing of Colonic Anastomoses

**DOI:** 10.1155/2017/1078062

**Published:** 2017-05-16

**Authors:** Malene Nerstrøm, Peter-Martin Krarup, Lars Nannestad Jorgensen, Magnus S. Ågren

**Affiliations:** ^1^Digestive Disease Center, Bispebjerg Hospital, University of Copenhagen, Bispebjerg Bakke 23, 2400 Copenhagen NV, Denmark; ^2^Copenhagen Wound Healing Center, Bispebjerg Hospital, University of Copenhagen, Bispebjerg Bakke 23, 2400 Copenhagen NV, Denmark

## Abstract

**Background:**

The mimetic compound OTR4120 may replace endogenous-degraded heparan sulfates that normally maintain the bioactivity of growth factors that are important for tissue repair. Herein, we investigated the effect of OTR4120 on the healing of normal colonic anastomoses.

**Methods:**

We evaluated the following two treatment groups of male Sprague Dawley rats (220–256 g): control-treated colonic anastomoses (*n* = 25) and OTR4120-treated colonic anastomoses (*n* = 25). We resected 10 mm of the left colon and then applied either saline alone (control) or OTR4120 (100 *μ*g/mL) in saline to the colonic ends before an end-to-end single-layer anastomosis was constructed and again on the anastomosis before the abdomen and skin were closed.

**Results:**

On postoperative day 3, the anastomotic breaking strengths were 1.47 ± 0.32 N (mean ± SD) in the control group and 1.52 ± 0.27 N in the OTR4120-treated animals (*P* = 0.622). We also found that the hydroxyproline concentration (indicator of collagen) in the anastomotic wounds did not differ (*P* = 0.571) between the two groups.

**Conclusions:**

Our data demonstrate that a single local application of OTR4120 intraoperatively did not increase the biomechanical strength of colonic anastomoses at the critical postoperative day 3 when the anastomoses are the weakest.

## 1. Introduction

Anastomotic leakage (AL) remains one of the most devastating complications after colorectal surgery despite improvements in surgical techniques and perioperative management. The incidence of AL is 3–7% after colocolonic anastomoses and 10–20% after colorectal anastomoses [1–3]. AL is associated with increased risks of morbidity, short-term mortality, permanent fecal diversion, cancer recurrence, and poor overall long-term survival [1, 4–7]. Many pharmaceuticals have been investigated but none have reached clinical usage for AL prophylaxis [8, 9].

Extracellular matrix (ECM) components play key roles in wound healing [10]. Previous experimental studies of uncomplicated anastomoses have demonstrated that there is substantial degradation of the submucosal ECM, which leads to lowered breaking strength, of experimental anastomoses three days postoperatively [11–16]. Thereafter, the anastomotic strength increases due to increased collagen biosynthesis [11–13].

Proteoglycans are a subset of complex ECM proteins with attached glycosaminoglycans and have unique properties. Heparan sulfates are glycosaminoglycans that protect heparin-binding growth factors, such as fibroblast growth factor-2 and transforming growth factor-*β* from proteolysis. Heparin-binding growth factors stimulate cell migration, proliferation, and differentiation [17–19]. Regenerating agents (RGTAs), including OTR4120 (OTR3, Paris, France), are synthetic heparan sulfate analogs derived from dextran [20]. These agents mimic the protective properties of endogenous heparan sulfates and enhance the in vivo bioavailability of heparin-binding growth factors [17, 18, 21]. These compounds may also directly prevent growth factor proteolysis by inhibiting serine proteinases such as neurophil elastase, plasmin, and cathepsin G [22–24]. RGTAs improve regeneration or healing after bone, muscle, tendon, skin, cornea, and colon insults [17, 18, 21, 25, 26].

Meddahi et al. [17] studied the effect of a RGTA in an animal model of anastomotic wound repair in the left colon. Their data showed that RGTA11 caused increased anastomotic bursting pressure 2 days after construction of the anastomoses but not after 4 or 7 days. Breaking strength is another biomechanical parameter for anastomosis integrity. Furthermore, the authors did not present biochemical metrics for wound healing [17].

The aim of this study was to investigate the impact of OTR4120 on the early healing of experimental colonic anastomoses under normal conditions by measuring the breaking strength and hydroxyproline (indicator for collagen). Because RGTAs have shown beneficial effects on skin wound healing [25, 26], we also investigated the effect of OTR4120 on the breaking strength of incisional skin wounds in the laparotomized animals.

## 2. Materials and Methods

The study was performed at the Panum Institute, University of Copenhagen, Copenhagen, Denmark. The study protocol was approved by the Animal Experiments Inspectorate of The Danish Ministry of Justice (2014-15-0201-00329). The ARRIVE guidelines were followed [27]. The experimental work was performed over a 3-week period. The investigators were blinded throughout the study and during data interpretation.

### 2.1. Animals

In this study, 50 male Sprague Dawley rats (Taconic Biosciences A/S, Lille Skensved, Denmark) weighing 220–256 g were used. The animals were housed in standard type III cages with a 12-hour light cycle and had free access to tap water and standard pellets. Two animals per cage were acclimatized for at least 10 days before surgery.

The rats were then transferred to individual cages and weighed on the day of surgery (day 0) and then again on days 1, 2, and 3. Their food consumption was also recorded on days 1, 2, and 3.

### 2.2. Sample-Size Calculation and Randomization

The sample size was calculated using data from one of our prior studies [15]. Three days postoperatively, the mean ± standard derivation (SD) anastomotic breaking strength was 1.19 ± 0.34 N in the control group and 1.54 ± 0.40 N in the experimental group. Based on a significance level (*α*) of 5% and a power (1-*β*) of 90%, we needed 24 animals in each group to detect an increase in anastomotic breaking strength from 1.19 N to 1.54 N (average SD: 0.37 N). We decided to include 25 animals in each group to account for 6% mortality.

The rats were randomized in blocks of 8 to control or OTR4120 treatment groups using a computer-generated sequence obtained from http://random.org.

### 2.3. Anesthesia and Analgesics

Anesthesia was introduced with isoflurane (Piramal Healthcare, Morpeth Northumberland, UK) 3.5%/O_2_ (1.5 L/min) and maintained with 2.0%/O_2_. Bupivacain (Marcaine®; AstraZeneca, Copenhagen, Denmark) 2 mg/kg was administered subcutaneously (s.c.) at the incisional site. The preoperative and postoperative analgesia was provided by per os 0.4 mg/kg buprenorphine (Temgesic®; Rickitt Benckiser, Berkshire, UK) mixed in hazelnut butter at 12-hour intervals.

### 2.4. Aseptic Surgical Procedures and OTR4120/Control (Saline) Administration

**Figure 1:**
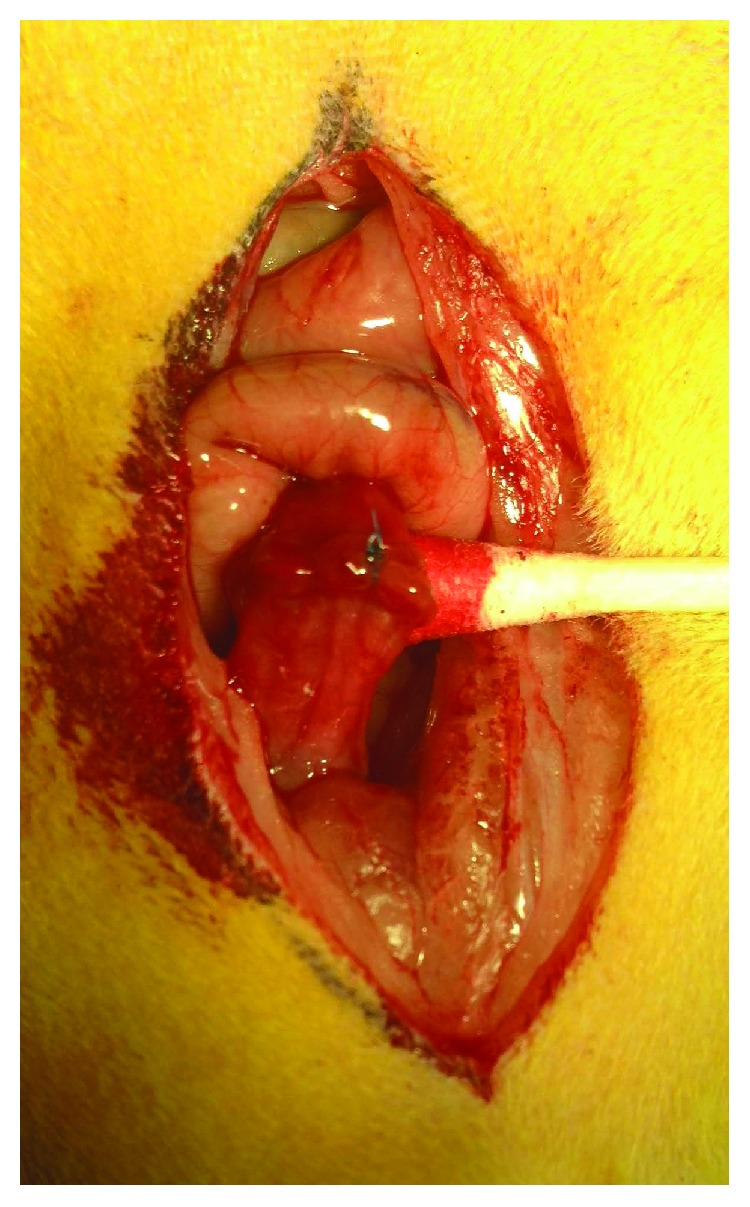
Colon anastomosis on day 0.

The abdomen was shaved, disinfected with 0.5% chlorhexidine gluconate in ethanol/isopropanol (Meda, Allerød, Denmark) and then covered with sterile surgical adhesive drape (Barrier®; Mölnlycke Health Care, Göteborg, Sweden). The peritoneal cavity was exposed through a 40 mm midline incision. A 10 mm segment of the left colon was resected with approximately 60 mm from the anus, and the fecal contents were removed. We applied 80 *μ*L of control (0.9% NaCl) or 80 *μ*L OTR4120 (100 *μ*g/mL) in saline corresponding to 8 *μ*g OTR4120 to each colonic end using a sterile low-density polyethylene Pasteur pipette (VWR, Radnor, PA, USA) based on the randomized allocation. An end-to-end single-layer anastomosis was constructed using 8 interrupted polypropylene monofilament 6/0 sutures (Premilene®; B. Braun Surgical, Jaén, Spain) as shown in Figure 1. We then applied 80 *μ*L of control or 80 *μ*L OTR4120 corresponding to 8 *μ*g OTR4120 to the sutured anastomosis. The total volume of control or OTR4120 solution applied per anastomosis was 240 *μ*L corresponding to 0 or 24 *μ*g OTR4120.

The abdominal muscles and the transverse fascia were closed using 8 interrupted polypropylene 4/0 sutures (Premilene). Before closing the skin, we applied 120 *μ*L of control or 120 *μ*L OTR4120 (100 *μ*g/mL) to each skin wound edge about 30 minutes after the laparotomy was performed [25, 26]. The skin was closed by 8 titanium clips (Appose ULC; Covidien, Mansfield, MA, USA). Then, an additional 240 *μ*L of control or 240 *μ*L OTR4120 were applied to the incisional skin wound. The total volume of control or OTR4120 solution applied per skin wound was 480 *μ*L corresponding to 0 or 48 *μ*g OTR4120.

After surgery, the rats were given 5 mL saline s.c. for rehydration.

### 2.5. Evaluation of Intraperitoneal Adhesions, Tissue Sampling, and Breaking Strength Determinations

On postoperative day 3, the rats were anesthetized and the skin clips were removed. The skin was resected én bloc (30 mm × 40 mm). The skin bloc was divided into two 15 mm (width) × 40 mm (length) strips with scissors. The strips contained the incision in the middle and were immersed in saline. The abdominal cavity was reopened and the degree of adhesion formation was assessed using the following scale: 0 = no adhesions; 1 = minimal adhesions, mainly between the anastomosis and the omentum; 2 = moderate adhesions occurring between the omentum and the anastomotic site and between the anastomosis and a loop of small bowel; and 3 = severe and extensive adhesions, including abscess formation [28]. The anastomosis was then freed of adhesions. A 40 mm-long colonic segment with the anastomosis in the middle was resected, and the fecal contents were gently evacuated. The anastomosis was examined macroscopically for signs of AL [29] and immersed in saline. The rats were then sacrificed by cervical dislocation.

The skin strips and anastomoses with sutures in situ were mounted in a materials testing machine (LF Plus; Lloyd Instruments, Bognor Regis, UK) with a 10 mm gap between the clamps. The tissues were stretched in the vertical direction at a constant speed of 10 mm/minute until rupture [14]. The testing was performed within 10 minutes of tissue excision. The breaking strengths in Newtons (N) were determined from the load-deformation curve (Nexygen software; Lloyd Instruments). The site of rupture was noted (anastomotic/incisional line or outside the anastomotic/incisional line). The mean breaking strength of the two skin strips from each animal was used in the subsequent data processing.

One tissue sample from each anastomotic wound was excised and placed in a cryogenic tube (Greiner Bio-One GmbH, Frickenhausen, Germany). The tissue was snap-frozen in liquid nitrogen and then transferred to −80°C until analysis for hydroxyproline as an indicator of collagen.

### 2.6. Hydroxyproline Determination

The tissue samples from the anastomotic wound were dried to constant weight (9.6 ± 2.6 mg, *n* = 49) at 100°C and hydrolyzed in 6 mol/L hydrochloric acid for 18 hours at 110°C. The samples were dried in a heating block and washed and dried with distilled water 3 times. The samples were then dissolved in 1 mL acetate-citrate buffer. Chloramine-T was added to the samples, and the solution was incubated at room temperature for 20 minutes. We then added p-dimethylaminobenzaldehyde-perchloric acid, and the samples were incubated for 25 minutes in a 60°C water bath [30]. The optical density was measured at 570 nm and the hydroxyproline concentration was derived from the standard linear curve for L-hydroxyproline (0–10 *μ*g/mL). The results are expressed as microgram (*μ*g) hydroxyproline per mg dry tissue weight. All measurements were performed in duplicate.

### 2.7. Statistical Analyses

All data were tested for normality using Kolmogorov-Smirnov's test and histograms. The breaking strength of the colonic anastomoses and the incisional skin wounds, colonic hydroxyproline concentration, and body weight were compared with the two-sided unpaired *t* test. These data are presented as the mean ± SD or mean ± SEM where indicated. The Mann-Whitney *U* test was used to compare the adhesion score and food consumption between the two groups. These results are presented as the median (interquartile range). A value of *P* < 0.05 was considered statistically significant. SPSS version 22.0 (IBM, Chicago, IL, USA) was used for the statistical analyses.

## 3. Results

One rat in the OTR4120 group died during recovery from anesthesia day 0.

### 3.1. Body Weights and Food Consumption

There was no difference in mean body weight between the control and OTR4120 groups on the day of surgery day 0 (Table 1). The mean body weights were significantly (*P* < 0.001) reduced in both groups from day 0 to day 3. However, the body weights did not differ significantly between the two groups on days 1, 2, or 3. In addition, postoperative food consumption did not differ significantly between the groups on days 1, 2, or 3 (Table 2).

### 3.2. Adhesion Formation

**Figure 2:**
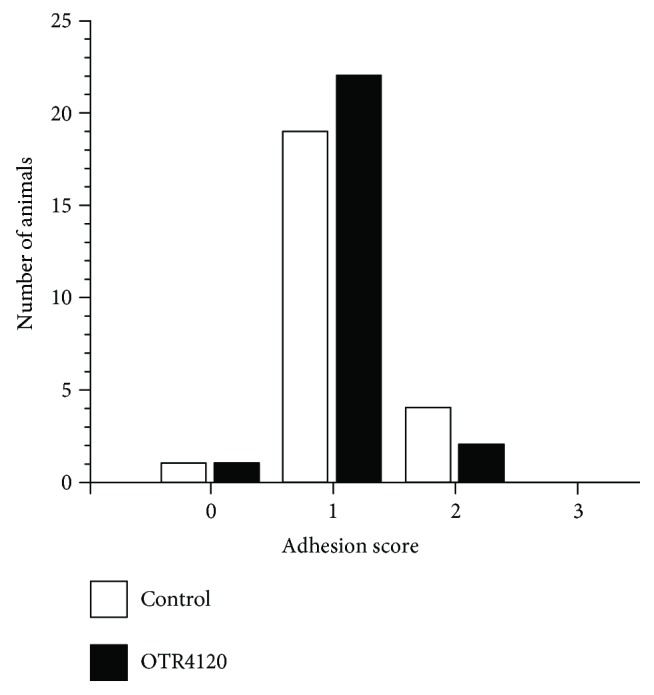
Intra-abdominal adhesion formation on postoperative day 3 in the control (open bars) and OTR4120 (black bars) groups [28].

The majority of rats had formed minimal intra-abdominal adhesions 3 days after surgery. The adhesion scores did not differ (*P* = 0.445) between the control and OTR4120 groups (Figure 2).

### 3.3. Anastomotic Wound Healing

**Figure 3:**
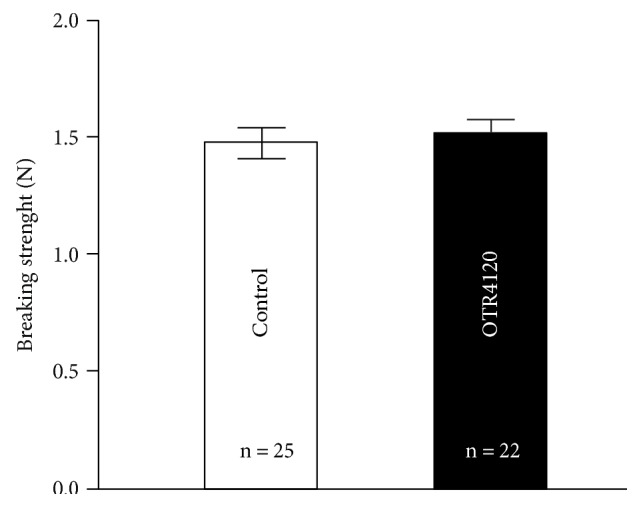
Breaking strength of colonic anastomoses on postoperative day 3 in the control (open bars) and OTR4120 (black bars) groups. Values are the mean ± SEM.

None of the rats showed signs of AL. There were two colonic anastomoses from the OTR4120 group that broke outside the anastomotic line and were therefore excluded from the primary statistical analysis. The breaking strength of the anastomoses in the control group was 1.47 ± 0.32 N (mean ± SD, *n* = 25) and 1.52 ± 0.27 N (*n* = 22) in the OTR4120 group (Figure 3). This difference was not significantly different (*P* = 0.622). Furthermore, an intention-to-treat analysis that included data from all animals did not show a significant difference in anastomotic breaking strength either between the two groups (*P* = 0.799).

**Figure 4:**
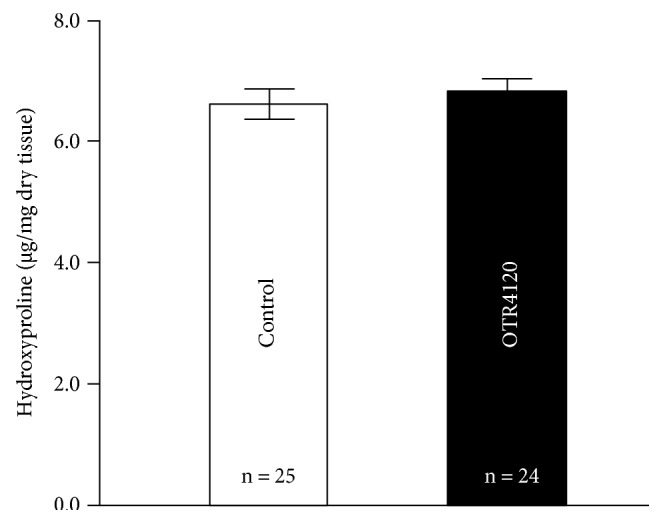
Hydroxyproline concentration of colonic anastomoses on postoperative day 3 in the control (open bars) and OTR4120 (black bars) groups. Values are the mean ± SEM.

The anastomotic wound hydroxyproline concentration in the control group was not significantly (*P* = 0.571) different from that of the OTR4120 group (Figure 4).

### 3.4. Breaking Strength of Incisional Skin Wounds

All the skin strips broke in the midline incision. Two wounds in the control group and one wound in the OTR4120 group were excluded due to technical failures. The breaking strength of wounds in the control group was 0.64 ± 0.15 N (mean ± SD, *n* = 23) and 0.67 ± 0.23 N (*n* = 23) in the OTR4120-treated skin wounds. These values were not significantly different (*P* = 0.737).

## 4. Discussion

The mimetic compound OTR4120 is thought to replace endogenous-degraded heparan sulfates that normally maintain the bioactivity of growth factors important for tissue repair [17, 18, 21, 25, 26]. In this study, we demonstrated that a single intraoperative topical application of OTR4120 did not increase the biomechanical strength of colonic anastomoses on postoperative day 3 in male rats.

We used data from the study by Krarup et al. to calculate the sample size [15]. The variability in anastomotic breaking strength measurements expressed as the coefficient of variation was 20% in the present study compared with 27% in the study by Krarup et al. [15]. Furthermore, post hoc analyses of our generated data revealed a 20% risk (type II error) of overlooking increased breaking strength of 0.25 N with OTR4120 treatment of the colonic anastomoses with 95% confidence. Taken together, these calculations suggest that the number of animals used in our study was sufficient to detect clinically significant differences in anastomotic breaking strength between the control and OTR4120 groups. It should be emphasized that the biomechanical analyses were performed on one time point only. We chose postoperative day 3 because numerous studies have shown that the anastomoses are the weakest [11–16] and therefore most probable to dehisce on postoperative day 3.

Our findings contrast with those of a previous study on RGTA11, which is an analog of OTR4120 [17]. In their study, the colonic ends were immersed in the RGTA11 solution (50 *μ*g/mL) for 2 minutes before the colonic anastomoses were constructed in 10 male rats per group. Our dosing protocol differed from that of Meddahi et al. [17]. First, we used twice the concentration of the OTR4120 solution (100 *μ*g/mL). Secondly, apart from applying the compound to the cut colonic ends before being sutured similar to Meddahi et al. [17], we also applied OTR4120 to the constructed anastomosis to maximize the therapeutic effect. Therefore, we judge it as unlikely that the differences in administration of OTR4120 would account for the lack of effect of OTR4120 on anastomotic breaking strength. Another possibility is the time-dependent decline of the bioactivity of OTR4120. Reapplication of OTR4120 to the anastomosis in the early postoperative period is, however, not a clinical option. However, anastomotic breaking strength and bursting pressure are two measures that reflect different phenomena and are not significantly correlated during early anastomotic wound healing [31]. This finding could explain the discrepancy with the study by Meddahi et al. [17] in anastomotic wound healing.

In the early postoperative period, the suture-holding submucosal collagen is responsible for the biomechanical strength of colonic anastomoses. Collagen degradation dominates over collagen biosynthesis during this period [16]. OTR4120 did not influence the collagen levels of the anastomoses.

The breaking strength of the incisional skin wounds on postoperative day 3 is the force required to disrupt the fibrin-fibronectin-rich but collagen-poor ganulation tissue formed between the wound edges [12, 13]. The treatment with OTR4120 did not enhance the biomechanical strength of the incisional skin wounds. This finding contrasts with the results from one previous study that reported beneficial effects on cutaneous wound healing including increased angiogenesis, granulation tissue formation, and epithelialization on postoperative days 3 and 7 in rats [25]. In addition, OTR4120 significantly improved the breaking strength of the full-thickness excisional wounds (15 mm in diameter day 0) at later time points (days 14 and 79) than that tested in our study [26]. In these studies, OTR4120 was also administered systemically (1 mg/kg weekly) in addition to the single topical application of 8 *μ*g OTR4120 at the time of wounding [25, 26]. Here, OTR4120 was not given systemically to avoid the increased risk of adverse effects.

The difference in the time between the initial injury and application of OTR4120 could theoretically have an effect on the wound-healing outcome [25, 26]. This lag period may be important because mimetic compounds are capable of reducing the initial release of matrix-bound growth factors, which may have a negative impact on wound healing [19]. Regardless, the delay between the initial skin injury and the application of OTR4120 was similar in the present study and in the studies of Tong et al. [25, 26].

## 5. Conclusion

This experimental study failed to demonstrate any beneficial effects of locally applied OTR4120 on the healing of colonic anastomoses and incisional skin wounds on postoperative day 3. No local or systemic adverse effects of OTR4120 were observed. However, the systemic administration of OTR4120 alone or in combination with topical OTR4120 application should be examined in future studies.

## Figures and Tables

**Table 1:** Animal body weights (g) of the two groups.

Group	Day			
	0	1	2	3
Control	276 ± 27	265 ± 26	266 ± 27	268 ± 26
*P*	0.517	0.387	0.560	0.829
OTR4120	282 ± 29	272 ± 28	270 ± 28	269 ± 28

Vales are the mean ± SD.

**Table 2:** Postoperative food consumption (g/day) in the two groups.

Group	Day		
	1	2	3
Control	5.5 (2.3–7.0)	8.3 (6.0–11.8)	10.8 (9.0–14.5)
*P*	0.413	0.059	0.316
OTR4120	3.3 (1.9–5.6)	5.0 (2.1–9.4)	10.3 (8.4–13.3)

Values are the median (interquartile range).

## References

[1] Krarup P. M., Jorgensen L. N., Andreasen A. H., Harling H. (2012). A nationwide study on anastomotic leakage after colonic cancer surgery. *Colorectal Disease*.

[2] Pommergaard H. C., Gessler B., Burcharth J., Angenete E., Haglind E., Rosenberg J. (2014). Preoperative risk factors for anastomotic leakage after resection for colorectal cancer: a systematic review and meta-analysis. *Colorectal Disease*.

[3] Danish Colorectal Cancer Group Nationwide database for cancer of the colon and rectum - yearly report. http://dccg.dk/03_Publikation/2013.pdf.

[4] Law W. L., Choi H. K., Lee Y. M., Ho J. W., Seto C. L. (2007). Anastomotic leakage is associated with poor long-term outcome in patients after curative colorectal resection for malignancy. *Journal of Gastrointestinal Surgery*.

[5] Choi H. K., Law W. L., Ho J. W. (2006). Leakage after resection and intraperitoneal anastomosis for colorectal malignancy: analysis of risk factors. *Diseases of the Colon and Rectum*.

[6] Peeters K. C., Tollenaar R. A., Marijnen C. A. (2005). Risk factors for anastomotic failure after total mesorectal excision of rectal cancer. *British Journal of Surgery*.

[7] Khan A. A., Wheeler J. M., Cunningham C., George B., Kettlewell M., Mortensen N. J. (2008). The management and outcome of anastomotic leaks in colorectal surgery. *Colorectal Disease*.

[8] Øines M. N., Krarup P. M., Jorgensen L. N., Ågren M. S. (2014). Pharmacological interventions for improved colonic anastomotic healing: a meta-analysis. *World Journal of Gastroenterology*.

[9] Nerstrøm M., Krarup P. M., Jorgensen L. N., Ågren M. S. (2016). Therapeutic improvement of colonic anastomotic healing under complicated conditions: a systematic review. *World Journal of Gastrointestinal Surgery*.

[10] Ågren M. S., Werthén M. (2007). The extracellular matrix in wound healing: a closer look at therapeutics for chronic wounds. *The International Journal of Lower Extremity Wounds*.

[11] Hendriks T., Mastboom W. J. (1990). Healing of experimental intestinal anastomoses. Parameters for repair. *Diseases of the Colon and Rectum*.

[12] Oxlund H., Christensen H., Seyer-Hansen M., Andreassen T. T. (1996). Collagen deposition and mechanical strength of colon anastomoses and skin incisional wounds of rats. *Journal of Surgical Research*.

[13] Syk I., Ågren M. S., Adawi D., Jeppsson B. (2001). Inhibition of matrix metalloproteinases enhances breaking strength of colonic anastomoses in an experimental model. *British Journal of Surgery*.

[14] Ågren M. S., Andersen L., Heegaard A. M., Jorgensen L. N. (2008). Effect of parenteral zinc sulfate on colon anastomosis repair in the rat. *International Journal of Colorectal Disease*.

[15] Krarup P. M., Eld M., Heinemeier K., Jorgensen L. N., Hansen M. B., Ågren M. S. (2013). Expression and inhibition of matrix metalloproteinase (MMP)-8, MMP-9 and MMP-12 in early colonic anastomotic repair. *International Journal of Colorectal Disease*.

[16] Ågren M. S., Andersen T. L., Mirastschijski U. (2006). Action of matrix metalloproteinases at restricted sites in colon anastomosis repair: an immunohistochemical and biochemical study. *Surgery*.

[17] Meddahi A., Benoit J., Ayoub N., Sezeur A., Barritault D. (1996). Heparin-like polymers derived from dextran enhance colonic anastomosis resistance to leakage. *Journal of Biomedical Materials Research*.

[18] Rouet V., Hamma-Kourbali Y., Petit E. (2005). A synthetic glycosaminoglycan mimetic binds vascular endothelial growth factor and modulates angiogenesis. *The Journal of Biological Chemistry*.

[19] Rouet V., Meddahi-Pelle A., Miao H. Q., Vlodavsky I., Caruelle J. P., Barritault D. (2006). Heparin-like synthetic polymers, named RGTAs, mimic biological effects of heparin in vitro. *Journal of Biomedical Materials Research. Part A*.

[20] Papy-Garcia D., Barbier-Chassefiere V., Rouet V. (2005). Nondegradative sulfation of polysaccharides. Synthesis and structure characterization of biologically active heparan sulfate mimetics. *Macromolecules*.

[21] Meddahi A., Bree F., Papy-Garcia D., Gautron J., Barritault D., Caruelle J. P. (2002). Pharmacological studies of RGTA(11), a heparan sulfate mimetic polymer, efficient on muscle regeneration. *Journal of Biomedical Materials Research*.

[22] Meddahi A., Lemdjabar H., Caruelle J. P., Barritault D., Hornebeck W. (1996). FGF protection and inhibition of human neutrophil elastase by carboxymethyl benzylamide sulfonate dextran derivatives. *International Journal of Biological Macromolecules*.

[23] Ledoux D., Papy-Garcia D., Escartin Q. (2000). Human plasmin enzymatic activity is inhibited by chemically modified dextrans. *The Journal of Biological Chemistry*.

[24] Ledoux D., Merciris D., Barritault D., Caruelle J. P. (2003). Heparin-like dextran derivatives as well as glycosaminoglycans inhibit the enzymatic activity of human cathepsin G. *FEBS Letters*.

[25] Tong M., Tuk B., Hekking I. M., Vermeij M., Barritault D., van Neck J. W. (2009). Stimulated neovascularization, inflammation resolution and collagen maturation in healing rat cutaneous wounds by a heparan sulfate glycosaminoglycan mimetic, OTR4120. *Wound Repair and Regeneration*.

[26] Tong M., Zbinden M. M., Hekking I. J., Vermeij M., Barritault D., van Neck J. W. (2008). RGTA OTR 4120, a heparan sulfate proteoglycan mimetic, increases wound breaking strength and vasodilatory capability in healing rat full-thickness excisional wounds. *Wound Repair and Regeneration*.

[27] NC3Rs Reporting Guidelines Working Group (2010). Animal research: reporting in vivo experiments: the ARRIVE guidelines. *The Journal of Physiology*.

[28] van der Ham A. C., Kort W. J., Weijma I. M., van den Ingh H. F., Jeekel J. (1991). Effect of fibrin sealant on the healing colonic anastomosis in the rat. *British Journal of Surgery*.

[29] Rehn M., Krarup P. M., Christensen L. H., Seidelin J. B., Ågren M. S., Syk I. (2015). GM6001 increases anastomotic leakage following colonic obstruction possibly by impeding epithelialization. *Surgical Infections*.

[30] Mirastschijski U., Johannesson K., Jeppsson B., Ågren M. S. (2005). Effect of a matrix metalloproteinase activity and TNF-alpha converting enzyme inhibitor on intra-abdominal adhesions. *European Surgical Research*.

[31] Ikeuchi D., Onodera H., Aung T. (1999). Correlation of tensile strength with bursting pressure in the evaluation of intestinal anastomosis. *Digestive Surgery*.

